# Cannabis Use and Dependence among French Schizophrenic Inpatients

**DOI:** 10.3389/fpsyt.2014.00082

**Published:** 2014-07-15

**Authors:** Michel Lejoyeux, Anne Basquin, Marie Koch, Houcine Embouazza, Florence Chalvin, Michaelle Ilongo

**Affiliations:** ^1^Department of Psychiatry and Addictive Medicine, Maison Blanche Hospital, Bichat-Claude Bernard Hospital, AP-HP, Paris Diderot University, Paris, France

**Keywords:** cannabis, addiction, dependence, alcohol, nicotine, compulsive buying, pathological gambling, alcohol dependence

## Abstract

**Background:** To assess the prevalence of cannabis use and dependence in a population of schizophrenic inpatients and to compare schizophrenics with and without cannabis consumption.

**Methods:** One hundred one schizophrenic patients were examined during their first week of hospitalization. They answered the PANNS scale of schizophrenia, the CAGE and the Fagerström questionnaire, and the DSM-IV-TR criteria for cannabis, alcohol, opiates, and nicotine use dependence were checked. We also assessed socio-demographic characteristics, the motive of cannabis consumption, and the number of cannabis joints and alcoholic drinks taken.

**Results:** The prevalence of cannabis consumption was 33.6% among schizophrenic inpatients. Schizophrenics consuming cannabis were younger than non-schizophrenics (33.3 vs. 44.7 years *p* < 0.0001), more often male (77 vs. 54%, *p* = 0.02) and had been hospitalized for the first time in psychiatry earlier (24.3 vs. 31.3 *p* = 0.003). Eighty-eight percent of cannabis consumers were dependent on cannabis. They were more often dependent on opiates (17 vs. 0%) and alcohol (32 vs. 7.4%, *p* = 0.001) and presented compulsive buying more often (48 vs. 27%, *p* = 0.04). Logistic regression revealed that factors associated to cannabis consumption among schizophrenics were cannabis dependence, male gender, pathological gambling, opiate dependence, number of joints smoked each day, and compulsive buying.

**Conclusion:** 33.6% of the schizophrenic patients hospitalized in psychiatry consume cannabis and most of them are dependent on cannabis and alcohol. Hospitalization in psychiatry may provide an opportunity to systematically identify a dependence disorder and to offer appropriate information and treatment.

## Introduction

Reported prevalence of substance use disorders in people suffering from schizophrenia ranges from 10 to 70% according to the assessment methods (1). Cannabis, the most frequently used illicit drug of the western world, is the preferred substance of patients with schizophrenia (2). Lifetime use of cannabis among schizophrenics is twice as high in the general population (1) and about one-quarter of schizophrenics have a lifetime cannabis use disorder (3). The use of psychoactive substances such as cannabis could be a form of self-medication in an attempt to mitigate symptoms and or to reduce side effects of the antipsychotic medication (2). Whether cannabis use is or not an etiological or precipitating factor of psychotic episodes is still in debate but cannabis seems to be at least a component cause in the development of schizophrenia (4).

Cannabis use is especially detrimental to people with schizophrenia (5, 6). It is associated with earlier onset of psychosis and with treatment-resistant forms of schizophrenia (7). Cannabis-using schizophrenics show higher relapse rates, have less access to non-pharmacological interventions, and need longer hospitalizations (8).

## Materials and Methods

Our work does not address the role of cannabis in the development of schizophrenia but is focused on the comparison between schizophrenics with and without cannabis consumption.

We try to answer the three following questions:

*Can we confirm the high prevalence of cannabis use and dependence in a population of French schizophrenic patients?* For this purpose we assessed the frequency of cannabis consumption and dependence among a population of schizophrenic inpatients.
*Do schizophrenic consuming cannabis present more severe social problems and are they more often hospitalized?* We compared socio-demographic characteristics and psychiatric history of schizophrenics with or without cannabis.
*Can cannabis consumption be understood as a self-medication for symptoms of schizophrenia or as a self-treatment for side effects of medications?* We tried to indirectly answer this question by asking patients about their motivation for cannabis consumption. We wanted to know if they used cannabis to alleviate anxiety or hallucinations or by boredom. In order to know if cannabis consumption is associated to more severe forms of schizophrenia, we compared severity of schizophrenia, anxiety, or depression among patients with and without cannabis consumption.

Due to its cross-sectional design, our work does not address the role of cannabis in the development of schizophrenia. It is focused on the effect of continuous use on symptoms of patients already suffering from schizophrenia.

The study was reviewed and approved by the ethical review board of the department of psychiatry and addictive medicine from Bichat (AP-HP) hospital. To ensure confidentiality, all identifying data were removed and all records were kept locked.

### Inclusion criteria

To be eligible, patients had to be between 18 and 65 years of age, able to understand the informed consent procedure and meet the DSM-IV-R criteria for schizophrenia. We proposed the questionnaire to all consecutive schizophrenic patients admitted in the same department of psychiatry during the 12 months of the study.

### Study design and recruitment

The study was conducted in the Bichat Maison Blanche Department of Psychiatry. This department is located in a Parisian University Hospital, which serves the North of Paris (France). Patients were not pre-selected and they strictly reflected patients usually admitted. In our practice, patients are only hospitalized when their behavior is a risk factor for themselves or for society. Hospitalized patients have often rejected regular care and pharmacotherapy. Patients at the time of the assessment were in regular treatment or had just started treatment. All patients were interviewed by a psychiatrist (Michaelle Ilongo) or a psychologist (Anne Basquin) trained to use the study instruments. Patients were assessed during their first week of stay in the department. Recruitment covered a 1-year period (2011). Assessment consisted in two sessions of 1 h with structured interviews. All patients could sustain their attention during the two sessions. Diagnosis of schizophrenia was confirmed with the Mini International Neuropsychiatric Interview (MINI) (9), which checks the DSM-IV diagnostic criteria. All patients included fulfilled the DSM-IV criteria for schizophrenia.

### Socio-demographics

We assessed demographic details (age, working status, marital status) and modalities of hospitalization through a specific and validated questionnaire (10).

### Psychiatric symptoms

Psychotic symptoms were studied with the Positive and Negative Symptom Scale (PANSS) (11, 12). Special attention was given to the items of the PANSS sub-scores of positive and negative symptoms. We assessed depression with the Montgomery–Asberg Depression Rating Scale (MADRS) (13). This scale is a 10-item clinician-rated scale measuring severity of depressive symptoms. Items are rated on a seven-point Likert scale (from 0 to 6) and the total score ranges from 0 to 60. The Hamilton Anxiety Rating Scale (14) provides an overall measure of global anxiety, including psychic (cognitive) and somatic symptoms.

### Substance use

Our first question was “did you smoke cannabis in the last 7 days prior to admission to the hospital?” Patients who answered positively to this question were included in the “cannabis +” group and compared to those who had not smoked during the last 7 days “cannabis −” group. To pursue the assessment of cannabis use disorders, we checked the DSM-IV-R criteria for cannabis abuse and dependence and used a specific questionnaire to quantify the number of joints smoked each day and the number of days per week in which patients smoked cannabis (15). In order to approach motives of cannabis consumption, we proposed to all patients six analogic visual scales. We asked them to score between 0 (I do not agree at all with this proposition) and 10 (I fully agree with this proposition) their answer to the following questions: I take cannabis to relax, to have a wild time, from force of habit, by boredom, to get stimulated, or to take away hallucinations.

Cigarette smoking was studied with the Fagerström questionnaire (16). This test contains four yes–no and two multiple-choice questions. The average score in randomly selected smokers is approximately 4–4.5. In samples of cigarette smokers seeking treatment, mean scores range from 5.2 to 6.3 (16). The DSM-IV-R criteria for nicotine dependence were also checked and we calculated the number of cigarettes smoked each day during the week before admission.

All patients answered the Cut–Annoyed–Guilty–Eye (CAGE) Opener questionnaire (17) to characterize their relation to alcohol. Item responses of the CAGE are scored 0 or 1. A total score of 2 or more is considered clinically significant. The quantity of drinks taken within a 24-h period during the last week was assessed with a specific questionnaire previously validated (15) – a drink being defined as the amount of alcohol (about 10 g) found in 300 ml of beer, 100 ml of wine, or 25 ml of whiskey. The number of drinking days per week in the month before the interview was also quantified. We measured the number of daily drinks from Monday to Thursday and the number of drinks at the end of the week (Friday to Sunday). We finished by checking the DSM-IV-R criteria for alcohol dependence. To identify other uses of illicit drug, we asked patients about their opiates and cocaine consumption and we checked the DSM-IV-R criteria of dependence for these two substances.

### Pathological gambling and compulsive buying

In addition to dependence to psychoactive substance, we also studied two frequent forms of behavioral addiction: pathological gambling and compulsive buying. We used a specific questionnaire (18) to assess compulsive buying. The 19 items (questions with yes–no answers) represent major basic features of compulsive buying: impulsivity, urges to shop and buy, emotions typically felt before, during, and after purchase, post purchase guilt and regrets, degree of commitment to short-term gratification, tangible consequences of buying, and avoidance strategies. Subjects with a score superior to nine are considered compulsive buyers. Pathological gambling was diagnosed according to the DSM-IV-R criteria. We checked the diagnostic criteria with a questionnaire previously validated (15).

### Analysis of data

For all data, we compared schizophrenic patients with or without cannabis consumption in the last 7 days (cannabis + and − groups). Differences between cannabis + and − groups were tested with Analysis of Variance (ANOVA) and with the Student’s *t*-test for quantitative parameters. We used the χ^2^ test to compare qualitative parameters. Predictive factors of the presence of cannabis consumption were also identified with multinomial regression analysis. The group without cannabis consumption was chosen as reference category for logistic regression. All statistical tests were two-tailed and a *p* value of 0.05 or less was considered to be significant.

## Results

We proposed the questionnaires to 145 consecutive schizophrenic patients admitted in the same department of psychiatry. Forty-four patients were not included in the study and 101 filled the questionnaires. Among the patients not included, 20 were unable to understand the study as a result of cognitive disabilities and 22 were too agitated or aggressive to fill the questionnaires. Thirty-four from the 101 patients (33.6%) included in the study had consumed cannabis in the week preceding the hospitalization.

Out of these 101 patients, 39 were female (Table 1). Mean age was 40.9 years. As expected, cannabis consumption was much more prevalent in males (77% of men among the cannabis + group vs. 54% in the cannabis − group). Patients from the cannabis + group were younger (33.3 vs. 44.7 years, *p* < 0.0001), more often male (77 vs. 54%, *p* = 0.02) and more often unemployed (91 vs. 72%, *p* = 0.03). The age of their first hospitalization in a psychiatric department was younger (24 vs. 31.3 years, *p* = 0.003).

**Table 1:** **Comparison of schizophrenic patients with and without cannabis consumption: socio-demographic characteristics, access to hospitalization, and duration of hospitalization**.

Variable	Cannabis +	Cannabis −	Total	Statistics
	*N* = 34	*N* = 67	*N* = 101	
Age (mean, SD)	33.3 (8)	44.7 (16)	40.9 (15)	Student *t* = 38, df = 99, ***p* ***<*** 0.0001**
Sex (*N* and % of women)	8 (23%)	31 (46%)	39 (38%)	χ^2^ = 4.9, df = 1, ***p* ***=*** 0.027**
Living alone (*N* and %)	22 (64%)	47 (70%)	69 (68%)	χ^2^ = 0.3, df = 1, *p* = 0.578
Working (*N* and %)	3 (9%)	19 (28%)	22 (21%)	χ^2^ = 5.05, df = 1, ***p* ***=*** 0.025**
**ACCESS TO HOSPITALIZATION (*N* AND %)**				
Ambulance	13 (38%)	27 (40%)	40 (39%)	χ^2^ = 8.7, df = 3, ***p* ***=*** 0.03**
Family	2 (6%)	17 (25%)	19 (18%)	
Police	8 (23%)	14 (21%)	22 (21%)	
Alone	11 (32%)	9 (20%)	20 (19%)	
Age of first hospitalization in psychiatry (mean, SD)	24 (6.6)	31.3 (13.4)	28.8 (11.9)	Student *t* = 3, df = 99, ***p* ***=*** 0.003**
Months of hospitalization in psychiatry (mean, SD)	9.2 (9.3)	17.1 (23.3)	14.4 (19)	Student *t* = 1.9, df = 99, *p* = 0.06

*Bold font indicates statistically significant difference*.

Unsurprisingly, as seen on Table 2, schizophrenics from the cannabis + group were more often dependent on cannabis (88 vs. 0%). They presented more often opiate dependence (17 vs. 0%), alcohol dependence (32 vs. 7.4%, *p* = 0.001). They drank more alcohol (5.4 vs. 1.3 drinks/day). The number of drinking days/week in the last month was higher in the cannabis + group (3.3 vs. 1.6 drinks *p* = 0.005). Their mean CAGE scores were higher (1.6 vs. 0.6, *p* < 0.001). None of the patients consumed cocaine. Prevalence of nicotine dependence was equivalent in the two groups (60 vs. 58%). The number of cigarettes smoked each day was also equivalent (10.7 vs. 9.6). Patients from the cannabis + group had higher scores of compulsive buying (5.4 vs. 3.1, *p* = 0.01) and presented diagnostic criteria of compulsive buying more often (48 vs. 27%, *p* = 0.04).

**Table 2:** **Comparison of addictive disorders among schizophrenic patients with and without cannabis consumption**.

Variable	Cannabis +	Cannabis −	Total	Statistics
	*N* = 34	*N* = 67	*N* = 101	
Alcohol consumption (*N* and %)	16 (55%)	18 (25%)	34 (33%)	χ^2^ = 84, df = 1, ***p* ***=*** 0.004**
Drinks of alcohol/day during week days (mean, SD)	2.64 (4.9)	0.43 (1.2)	1.1 (3.1)	Student *t* = −3.5, df = 99, ***p* ***=*** 0.001**
Drinks of alcohol/day at week-ends (mean, SD)	2.4 (3.8)	0.53 (1.3)	1.1 (2.6)	Student *t* = −3.7, df = 99, ***p* ***<*** 0.0001**
Number of drinking days/week (mean, SD)	3.3 (3)	1.6 (2.6)	2.2 (2.8)	Student *t* = −2.8 ***p* ***=*** 0.005**
Alcohol dependence (*N* and %)	11 (32%)	5 (7.4%)	16 (15%)	χ^2^ = 10.4, df = 1, ***p* ***=*** 0.001**
CAGE score (mean, SD)	1.6 (1.1)	0.6 (1.1)	1.1 (1.2)	Student *t* = −4, df = 99, ***p* ***<*** 0.001**
Nicotine dependence (*N* and %)	21 (60%)	39 (58%)	60 (59%)	χ^2^ = 0.23, df = 1, *p* = 0.6
Cigarettes/day (mean, SD)	10.7 (9.5)	9.6 (8.7)	10.1 (8.9)	Student *t* = −0.55, df = 99, *p* = 0.57
Fagerström score (mean, SD)	5.5 (2.2)	5.2 (2.1)	5.4 (2.2)	Student *t* = −0.2, df = 99, *p* = 0.7
Joints/day (mean, SD)	3.35 (3.8)	0.05 (0.2)	1.1 (2.7)	Student *t* = −7, df = 99, ***p* ***<*** 0.0001**
Days/week smoking cannabis (mean, SD)	4.4 (1.1)	0.28 (0.6)	1.6 (2.1)	Student *t* = −22.6, df = 99, ***p* ***<*** 0.0001**
Years of cannabis consumption (mean, SD)	12 (6.6)	0.5 (1.5)	4.6 (6.6)	Student *t* = −14.6, df = 99, ***p* ***<*** 0.0001**
Cannabis dependence (*N* and %)	30 (88%)	0	30 (29%)	χ^2^ = 84, df = 1, ***p* ***<*** 0.0001**
Opiate dependence (*N* and %)	6 (17%)	0	6 (5%)	χ^2^ = 12.5, df = 1, ***p* ***<*** 0.0001**
Pathological gambling (*N* and %)	4 (11%)	2 (2.9%)	6 (5%)	χ^2^ = −0.5, df = 1, *p* = 0.56
Scores of compulsive buying (mean, SD)	5.4 (4.8)	3.1 (3.8)	3.9 (4.1)	Student *t* = −2.5, df = 99, ***p* ***=*** 0.01**
Diagnostic of compulsive buying (*N* and %)	16 (48%)	18 (27%)	33 (32%)	χ^2^ = 41, df = 1, ***p* ***=*** 0.04**

*Bold font indicates statistically significant difference*.

We did not see (Table 3) differences between the cannabis + and − group in terms of symptoms of schizophrenia as assessed with the PANNS. Global functioning was equivalent in the two groups and schizophrenics smoking cannabis were not more depressed or anxious than non-smokers. The most frequent motives for cannabis consumption (Table 4) were “to relax,” “to have a wild time,” and “from force of habit.”

**Table 3:** **Comparison of psychiatric symptoms among schizophrenic patients with and without cannabis consumption**.

Variable	Cannabis +	Cannabis −	Total	Statistics
	*N* = 34	*N* = 67	*N* = 101	
PANNS total score (mean, SD)	33.4 (9.7)	33.4 (10.1)	33.4 (9.5)	Student *t* = −0.001, df = 99, *p* = 0.9
PANNS sub-score of positive symptoms (mean, SD)	20.1 (7.7)	22.2 (7.4)	21.5 (7.5)	Student *t* = 1.2, df = 99, *p* = 0.2
PANNS sub-score of negative symptoms (Mean, SD)	17.5 (6.2)	18.1 (8.3)	17.9 (7.5)	Student *t* = 0.35, df = 99, *p* = 0.72
Global assessment of functioning (mean, SD)	49 (11)	51 (13)	50.4 (12.4)	Student *t* = 0.8, df = 99, *p* = 0.4
MADRS score of depression (mean, SD)	11.4 (8.3)	9.2 (8.1)	9.9 (8.2)	Student *t* = −1.2, df = 99, *p* = 0.2
Hamilton score of anxiety (mean, SD)	11.5 (7.5)	10.5 (9.3)	10.8 (8.6)	Student *t* = 0.5, df = 99, *p* = 0.56

**Table 4:** **Motives of cannabis consumption (mean scores and SD)**.

To relax: 6.2 (3.4)
To have a wild time: 4.4 (3.7)
From force of habit: 3.8 (3.5)
By boredom: 3.2 (3.1)
To get stimulated: 2.7 (3.2)
To take away hallucinations: 2.5 (3.7)

Logistic regression (Table 5) showed that cannabis consumption was significantly associated to the diagnostic of cannabis dependence, male gender, pathological gambling, opiates dependence, number of joints smoked each day, and compulsive buying.

**Table 5:** **Logistic regression assessing factors associated to cannabis consumption**.

Variable	Odd ratio	Range	*p*
Cannabis abuse or dependence	235	5–5700	0.005
Male gender	2.9	0.3–27	0.3
Pathological gambling	1.7	0.6–47	0.7
Opiates dependence	1.66	0.5–98	0.06
Joints/day	1.52	1–2	0.04
Compulsive buying	1.5	0.6–47	0.7

*Reference category: schizophrenia with cannabis consumption likehood ratio = 131, df = 12, *p* < 0.0001*.

## Discussion

### Main findings

#### Cannabis consumption is frequent among French schizophrenic inpatients

Cannabis was the second psychoactive substance most often consumed (34 patients, 33.6%) after nicotine and at the same rate as alcohol in our population of patients. The other psychoactive substances consumed were nicotine (59%), alcohol (33%), and opiates (6, 5%).

#### Socio-demographic characteristics of schizophrenics smoking cannabis are specific

Schizophrenics from the cannabis + group were younger. They had been hospitalized for the first time in psychiatry at a younger age. Only a prospective study could confirm that cannabis consumption induces an earlier apparition of psychotic symptoms. Another difference between the cannabis + and − groups concerns the sex ratio. Proportion of male was higher in the cannabis + group.

#### Cannabis consumption is often part of a poly addictive disorder

Cannabis consumption is highly associated to cannabis dependence. Eighty-eight percent of patients consuming cannabis were dependent of this substance. The number of joints smoked by day is also associated in the logistic regression analysis. In the cannabis + group, alcohol consumption was higher and more frequent in week days and at week-ends. Even behavioral dependence like compulsive buying is more frequent in schizophrenics who smoke cannabis. This observation of an association between compulsive buying and chemical addictions was demonstrated among patients examined in emergency or in depressed patients (18). It had never been shown in schizophrenics. We observed another association between cannabis consumption and another behavioral dependence: pathological gambling. At our knowledge, this association has not been previously reported. These two associations could be an incitation to more systematically assess behavioral addictions among schizophrenics who smoke cannabis. Nicotine was the only psychoactive substance consumed in an equivalent proportion in the cannabis + and − groups. The level of consumption of nicotine was however high in both groups (60 and 58% in the cannabis + and − group, respectively).

#### Cannabis consumption is not associated to higher severity of psychiatric symptoms

We did not find a difference in psychiatric symptoms between the cannabis + and − groups. Patients from both groups had equivalent levels of depression, anxiety, negative, and positive symptoms of schizophrenia. Since our study is cross-sectional, we cannot conclude to an absence of effect of these symptoms. We can only observe the lack of difference. Study of motives for smoking does not corroborate the hypothesis of cannabis use as a “self-treatment” of psychotic symptoms. It does not incite, in clinical practice to authorize cannabis consumption as a way of alleviating negative and/or positive symptoms of schizophrenia. The motive of cannabis smoking less often cited by patients is “to take away hallucinations” and the most often chosen is “to relax.” A limitation of the interpretation of this result is related to the fact that all patients, whether or not they were cannabis smoker, were hospitalized and treated. Hypothesis can be raised that their treatments may have hidden differences in psychotic, depressive, or anxious symptoms.

### Limitations of the study

One of the principal limitations of the study is the exclusion of patients unable to answer the questionnaire because of the severity of their psychiatric state. Patients most severely affected were thus not included and their relation to cannabis was not identified. Another limitation is linked to the method of the study. Due to its cross-sectional design, our study does not address the important question of the potential role of cannabis in the apparition of schizophrenia. Only a prospective study could tell us if cannabis has or not a direct impact on schizophrenia severity and evolution. Our study, in addition, relies only on self-declarations of the patients. No biological assessment was performed to confirm declarations of patients regarding psychoactive substances consumption. Previous works (19) showed that patients with schizophrenia underreport their consumption of alcohol and cannabis. Up to 52% of patients deny their consumption when their declarations are compared to biological measures. We tried however to reduce this bias of underreporting by using standardized questionnaires. Moreover, our observed prevalence rate for cannabis consumption is equivalent to those from other studies, which confirmed clinical assessment with a biological measure (2). In spite of all its limitations, this study is the only work that has simultaneously assessed symptoms and severity of schizophrenia, anxiety, depression, and all dependence disorders to psychoactive substance and behavior (pathological gambling, compulsive buying).

In the naturalistic setting of our study most people who use cannabis make joints that contain cannabis and tobacco in differing compositions and of different strength (2). Besides Delta-9-Tetra-Hydro-Cannabinol (Δ-9-THC), the component that may have psychogenic properties, cannabis contains more than 60 other psychoactive substances, some of which possibly interfere with or even antagonize each other. We thus compare patients with different levels of intoxications even when they take the same number of joints by day. Another limitation concerns our study of nicotine consumption. We assessed nicotine dependence and nicotine consumption. We did not identify patients with nicotine consumption without dependence. The generalization of our results must also be limited since they are drawn from a relatively small population (101 subjects), all examined in the same hospital from the north of Paris.

#### Implication of the results for prevention and public health

The main finding of our research is the high prevalence of cannabis use and dependence among schizophrenics and the fact that this disorder is often unrecognized and under-treated. This observation is an incitation to systematically assess cannabis dependence and other forms of chemical and behavioral dependence in patients hospitalized for schizophrenia. In the majority of cases, dependence disorder is not recognized and patients neither receive a treatment nor information for their comorbid addiction. In the worse cases, cannabis consumption is tolerated by psychiatric departments, which wrongly consider this substance as a way for patients to cope with their disease and medication side effects. Schizophrenic patients must be directly asked about their relation to cannabis, and if the diagnosis of abuse or dependence is established, addictive disorders and co-existing psychiatric disorders should be addressed synchronously rather than sequentially (20). None of the patients identified as cannabis dependent had asked for specific help in the domain of addiction before being diagnosed by our systematic intervention. If they had not been assessed, they would have left the department of psychiatry with no treatment or information on the possibilities of help.

The time of hospitalization in psychiatry provides the only opportunity to receive information (5) and to begin to benefit from adequate treatment. It could be a teachable moment for a proposition of treatment for alcohol dependence regularly associated to cannabis dependence. Matthew Koola et al. (5) showed in a population of schizophrenics from Baltimore that 95.8% of them used quitting strategies to maintain abstinence from cannabis. The most commonly used quitting strategies were getting rid of cannabis (72%), getting rid of cannabis paraphernalia (67%), stopped association with people who smoke cannabis (62%) and no longer frequenting places where cannabis was smoked (61%). A brief motivational help on their cannabis consumption could also be proposed to patients with equivalent modalities of those proposed for alcohol dependents (20, 21).

## Conclusion

Cannabis consumption is frequent among schizophrenic patients hospitalized in psychiatry (33.6%). Typical schizophrenic patient at risk for cannabis consumption and dependence is a young man, who smokes, drinks, and is unemployed. Cannabis consumption is often associated to dependence to cannabis and other psychoactive substances (alcohol, opiates) at the exception of nicotine dependence equivalent among cannabis smokers and non-smokers. Behavioral dependence like compulsive buying is also more frequent among schizophrenics smoking cannabis. Systematic assessment and recognition of cannabis consumption must be performed during hospitalization of schizophrenic patients. Early assessment of cannabis dependence enables a better evolution of both psychotic and addictive disorders. Hospitalization may provide an opportunity to offer information and treatment for this frequent addictive disorder. Further prospective studies are needed to confirm the impact of cannabis consumption and use disorders on the evolution of schizophrenia.

## Conflict of Interest Statement

The authors declare that the research was conducted in the absence of any commercial or financial relationships that could be construed as a potential conflict of interest.
